# Quantification of Xylanolytic and Cellulolytic Activities of Fungal Strains Isolated from *Palmaria palmata* to Enhance R-Phycoerythrin Extraction of *Palmaria palmata*: From Seaweed to Seaweed

**DOI:** 10.3390/md21070393

**Published:** 2023-07-05

**Authors:** Yoran Le Strat, Margaux Mandin, Nicolas Ruiz, Thibaut Robiou du Pont, Emilie Ragueneau, Alexandre Barnett, Paul Déléris, Justine Dumay

**Affiliations:** Nantes Université, Institut des Substances et Organismes de la Mer, ISOMer, UR 2160, F-44000 Nantes, France; yoranlestrat@orange.fr (Y.L.S.); nicolas.ruiz@univ-nantes.fr (N.R.); paul.deleris@univ-nantes.fr (P.D.)

**Keywords:** marine fungi, cellulase, xylanase, macroalgal degradation, R-PE extraction

## Abstract

R-phycoerythrin (R-PE) can be enzymatically extracted from red seaweeds such as *Palmaria palmata*. This pigment has numerous applications and is notably known as an antioxidant, antitumoral or anti-inflammatory agent. Enzymes secreted by *P. palmata* associated fungal strains were assumed to be efficient and adapted for R-PE extraction from this macroalga. The aim of the present study was to quantify both xylanolytic and cellulolytic activities of enzymatic extracts obtained from six *Palmaria palmata* derived fungal strains. Degradation of *P. palmata* biomass by fungal enzymatic extracts was also investigated, focused on soluble protein and R-PE extraction. Enzymatic extracts were obtained by solid state fermentation. Macroalgal degradation abilities were evaluated by measuring reducing sugar release using DNS assays. Soluble proteins and R-PE recovery yields were evaluated through bicinchoninic acid and spectrophotometric assays, respectively. Various enzymatic activities were obtained according to fungal isolates up to 978 U/mL for xylanase and 50 U/mL for cellulase. Enzymatic extract allowed high degrading abilities, with four of the six fungal strains assessed exhibiting at least equal results as the commercial enzymes for the reducing sugar release. Similarly, all six strains allowed the same soluble protein extraction yield and four of them led to an improvement of R-PE extraction. R-PE extraction from *P. palamata* using marine fungal enzymes appeared particularly promising. To the best of our knowledge, this study is the first on the use of enzymes of *P. palmata* associated fungi in the degradation of its own biomass for biomolecules recovery.

## 1. Introduction

Seaweeds are traditionally consumed in Asian countries for a long time [1,2]. Their wide use in Europe is more recent (consumption of seaweeds as vegetables and condiments is authorized from 1990 in France) and is gradually increasing from several decades, notably because of an increasing demand of healthy food, with low ecological impact [1,2,3].

Algae may be the most diverse group of organisms alive, representing one third of the total divisions of the plant kingdom [4]. Among macroalgae, red seaweeds (Rhodophyceae) are characterized by the highest specific diversity, representing 45% of total seaweed species, followed by brown (Phaeophyceae) and green ones (Chlorophyceae) [5,6]. Red seaweeds are also the main macroalgal group in terms of commercial values [6] and in terms of secondary metabolite production [7]. Hydrocolloids of red seaweeds, being agars and carrageenans, are not only used in food but also in pharmaceutics [2,4]. Indeed, in addition to the useful thickening and gelling properties in formulation, these macroalgal sulphated polysaccharides can be characterized by biological activities such as antiviral ones [8,9]. Besides hydrocolloids, red seaweeds are also widely known to synthesize a great diversity of bioactive compounds with biotechnological interests and health-related properties, such as pigments, lipids, or polyphenols as examples [2,8,10,11,12].

Red seaweed would be more promising than green and brown macroalgae according to their protein contents [13,14,15,16,17]. The red seaweed *P. palmata* is one of the best macroalgal protein producers with a protein fraction representing up to 35% of the dry weight [1,14,18,19]. Quantitatively, these protein levels can be higher than the ones observed in soybean [14,18]. From a qualitative point of view, essential amino acids represent 46% of total amino acids from *P. palmata* and the composition can be compared with that of soybean or eggs [14,15,18]. However, the access and the digestibility of these proteins are limited by polysaccharides such as xylan in *P. palmata*, which are antinutritional [15,18]. One of the proteins of interest synthesized by Rhodophyceae, including *P. palmata*, is the phycoerythrin pigment, belonging to phycobiliproteins and used notably as an immunofluorescence dye [14].

Phycobiliproteins are water soluble algal pigments which can represent 20% of algal dry weight and which can be separated between phycoerythrin (PE), phycocyanin (PC), allophycocyanin (APC) and phycoerythrocyanin (PEC) [18,20,21,22,23]. These phycobiliproteins are composed of two different subunits, α and β. Only PE possesses another γ subunit in its central cavity, which allows a higher structural stability [18,23,24,25]. R-PE is the major phycobiliprotein in red seaweeds and is responsible for their coloration [18,26].

Phycobiliproteins are not harmful to humans when ingested or applied to the external surface; they are widely used in food and cosmetics and as a photosensitizer for tumor treatment [23]. More specifically, R-PE is characterized by an important biotechnological interest. It can be used as a pink colorant, particularly in the food industry, and as fluorescent probe in several technologies such as flow cytometry, fluorescent immunoassays or immunophenotyping [14,18,27,28]. R-PE was also characterized by several bioactivities such as antitumoral, anti-ageing, anti-inflammatory, antioxidant, antidiabetic, antiparasitic, immunosuppressive and antihypertensive ones [9,16,17,18,22,24,26]. It also has potential in application in solar cells [27]. R-PE potential in nanotechnologies is also highlighted by a study exhibiting the great enhancement of its electrical conductivity with an association of Ag0 nanoparticles in the R-PE channel [29]. The price of R-PE generally varies between USD 180 and USD 250/mg and it depends principally on the R-PE purity [24].

However, the R-PE uses are still limited to small scales by several issues about its extraction, purification and conservation but various strategies are currently in development to cope with these problems. One of the biggest issues for the R-PE extraction, which also concerns all soluble proteins and metabolites of interest from macroalgae, is the presence of cell wall polysaccharides having a retention effect and intracellular polysaccharides exhibiting ionic interactions with proteins [28,30,31,32,33]. In *P. palmata*, these polysaccharides are mostly xylan and cellulose, in a lower amount [18,19,34,35]. The conventional process of R-PE extraction consisted of osmotic shock or liquid nitrogen grinding but these technics were time consuming, not very efficient, and not applicable to the industrial scale due to their cost [18,24,36]. Currently, the most widespread technique for R-PE extraction is the enzyme-assisted extraction (EAE) [18,28]. EAE is a particularly promising process, widely used in macroalgal metabolite recovery, as an eco-friendly process, with high yield of products and a good quality [11,37]. The EAE process allows the extraction of many inaccessible compounds due to the polysaccharides’ cell wall and can be used in association with other extraction approaches such as a microwave or an ultrasound [11,18,31].

Several studies about enzymatic assisted extraction use on the R-PE recovery from *P. palmata* were performed [14,18,19,24,26,32]. R-PE enzyme assisted extraction has also been studied in other red seaweeds such as *Gracilaria gracilis* [38], *Grateloupia turuturu* [30] or *Gelidium pusillum* [39]. The cell walls of red seaweeds are composed of various polysaccharides which differ from one species to another and are responsible for the use of adapted enzymes in order to solubilize compounds of interest such as R-PE and other proteins [24,38].

It has been previously shown that the use of both xylanase and cellulase would allow an optimal extraction compared with the use of only one enzyme [24,32]. This synergistic effect seems not to be characteristic of all cellulases and xylanases, and it would depend on the biological origin of enzymes used for extraction [14].

Moreover, even if commercial enzymes have been successfully studied for the R-PE extraction from *P. palmata* by degrading its cell wall, the importance of the enzymatic specificity for the substrate has previously been mentioned. Indeed, EAE seems to be particularly efficient using algal-specific enzymes [37]. Marine enzymes including cellulases would be particularly industrially promising compared with terrestrial ones by exhibiting stabilities toward several physicochemical parameters such as pressure, pH, temperature, salinity, metals and chemicals or solvents [40]. Among marine enzyme producers, fungi would be particularly interesting, compared with bacteria, by excreting enzymes in the extracellular medium and exhibiting higher enzymatic activities [41,42,43], as well as having several abilities such as crystalline cellulose degradation [40].

The present study documents six fungal strains isolated from *P. palmata* and quantitatively assayed for xylanase and cellulase activities. Enzymatic extracts obtained from those selected strains were also tested on the R-PE recovery from *P. palmata* and compared with conventional commercial used enzymes.

## 2. Results

### 2.1. Quantifications of Xylanase and Cellulase Activities

**Figure 1:**
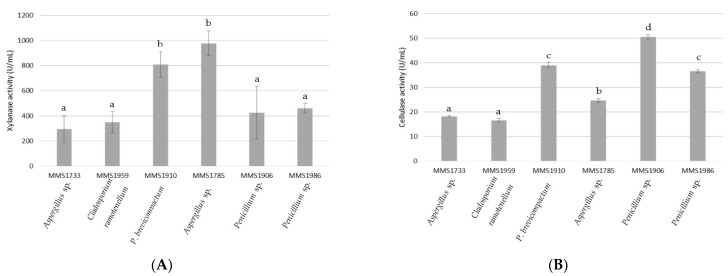
Xylanase (**A**) and cellulase (**B**) activity in U/mL (1 U = 1 µmol/min) of the six tested fungal strains. Letters represent clustering of non-significantly different values according to a post-hoc pair-wise Student test.

**Figure 2:**
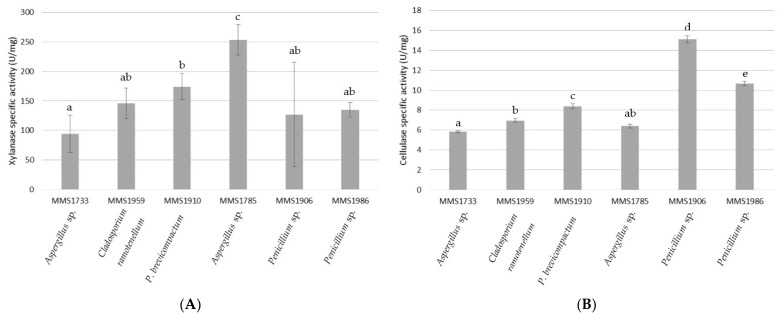
Xylanase (**A**) and cellulase (**B**) specific activity in U/mg (1 U = 1 µmol/min) of the six tested fungal strains. Letters represent clustering of non-significantly different values according to post-hoc pair-wise Student’s test.

The six fungal strains, *Aspergillus* sp. MMS1733, *Aspergillus* sp. MMS1785, *Penicillium* sp. MMS1906, *Penicillium brevicompactum* MMS1910, *Cladosporium ramotenellum* MMS1959, and *Penicillium* sp. MMS1986 were cultured on the *P. palmata* biomass based medium. Secretomes were used for enzymatic activity measurements and degradation of fresh *P. palmata* thalli. Results of cellulase and xylanase enzymatic and specific activities were represented in Figure 1 and Figure 2, respectively.

Regarding the xylanase activity assays (Figure 1A), both *Aspergillus* sp. MMS1785 and *Penicillium brevicompactum* MMS1910 exhibited xylanase activities significantly different and at least 2.1 and 1.8 times higher than the four other isolates, respectively (*p*-value < 0.01). Standard deviations for xylanase activities were more important than considering cellulase ones. Then, no clear distinction could be made between *Aspergillus* sp. MMS1733, *Penicillium* sp. MMS1986, *Penicillium* sp. MMS1906 and *Cladosporium ramotenellum* MMS1959 according to their xylanase activities which ranged between 295.4 and 461.8 U/mL. The two highly active isolates *P. brevicompactum* MMS1910 and *Aspergillus* sp. MMS1785 exhibited xylanolytic activities at 809.7 and 978 U/mL, respectively.

Regarding cellulase assays, the three best cellulolytic isolates belonged to the *Penicillium* genus with activities higher than 35 U/mL, and two intermediate strains belonged to the *Aspergillus* genus with cellulase activities ranging from 24.7 to 36.5 U/mL (Figure 1B). The isolate characterized by the lowest cellulase activity was the one assigned as *C. ramotenellum* (16.6 U/mL). Clear significant distinctions could be made between these three highly cellulolytic strains (*Penicillium* sp. MMS1906, *P. brevicompactum* MMS1910 and *Penicillium* sp. MMS1986) and the three other strains (*C. ramotenellum* MMS1959, *Aspergillus* sp. MMS1733 and *Aspergillus* sp. MMS1785) (*p*-value < 0.001). The three *Penicillium* isolates with the highest cellulase activities were also characterized as endophytic. Nonetheless, the location of the three other strains could not be determined as neither endophytic nor epiphytic. The best cellulolytic strain was *Penicillium* sp. MMS1906 with an activity at 50.5 U/mL.

The specific activities of all the six strains were measured for both cellulase and xylanase (Figure 2). They ranged from 94.4 (*Aspergillus* sp. MMS1733) to 253.7 (*Aspergillus* sp. MMS1785) U/mg for xylanase and from 5.8 (*Aspergillus* sp. MMS1733) to 15.1 (*Penicillium* sp. MMS1906) U/mg for cellulase.

Based on those results, no clear and significant distinction could be made between xylanase specific activities of *Aspergillus* sp. MMS1733, *C. ramotenellum* MMS1959, *P. brevicompactum* MMS1910, *Penicillium* sp. MMS1906 and *Penicillium* sp. MMS1986 strains, with values ranging from 94.4 and 174.2 U/mg (Figure 2A). Nevertheless, the *Aspergillus* sp. MMS1785 strain stayed the best xylanolytic one with a specific activity reaching 253.7 U/mg, significantly higher than xylanase specific activities of other strains.

Regarding cellulase, the three strains *Aspergillus* sp. MMS1733, *C. ramotenellum* MMS1959 and *Aspergillus* sp. MMS1785 exhibited the lowest specific activities, ranging from 5.8 to 6.9 U/mg (Figure 2B). The three other strains, *P. brevicompactum* MMS1910, *Penicillium* sp. MMS1986 and *Penicillium* sp. MMS1906 were characterized by cellulolytic specific activities significantly different between themselves, and to the three previously mentioned strains. They exhibited cellulase specific activity at 8.4, 10.7 and 15.1 U/mg, respectively. The strain *Penicillium* sp. MMS1906 was then characterized by both highest enzymatic and specific activities regarding cellulase.

Comparing both cellulase and xylanase (enzymatic and specific activities), it appeared that all fungal strains clearly exhibited higher xylanase than cellulase activity (Figure 1 and Figure 2). The strain *Penicillium* sp. MMS1906 was characterized by a xylanolytic activity 8.4 times higher than the cellulolytic one, being the lowest gap between cellulolytic and xylanolytic activity compared with other isolates. The greatest difference between cellulolytic and xylanolytic activities was detected with *Aspergillus* sp. MMS1785, with a xylanolytic activity 39.6 times higher than cellulolytic one.

### 2.2. Reducing Sugars, R-PE and Total Proteins Amounts from P. palmata Lysates

**Figure 3:**
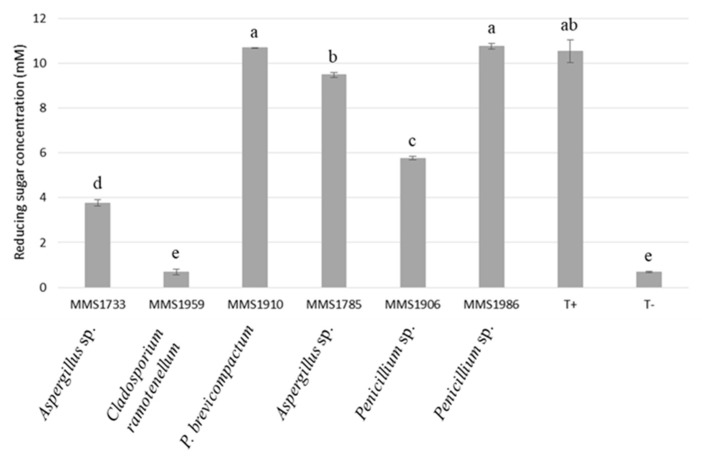
Reducing sugar concentration (mM) in the algal lysates after fungal enzymatic digestion. Negative control (T-) corresponded to an algal lysate obtained without enzyme added in the reactor, while positive control (T+) corresponded to an algal lysate obtained with addition of commercial xylanase in the reactor. Letters represent clustering of non-significantly different values according to post-hoc pair-wise Student’s test.

Reducing sugar extraction ranged between 0.69 mM and 10.76 mM according to the six fungal isolates (Figure 3). Enzymatic extracts from strains *P. brevicompactum* MMS1910 and *Penicillium* sp. MMS1986 permitted them to reach similar concentrations of reducing sugar in algal filtrate at 10.69 and 10.76 mM, respectively. They were significantly different to reducing sugar concentrations obtained with the other fungal strains (*p*-value < 0.05). *Aspergillus* sp. MMS1785, *Penicillium* sp. MMS1906, *Aspergillus* sp. MMS1733 and *C. ramotenellum* MMS1959 were characterized by a respective decrease regarding reducing sugar release from algal biomass, at 9.48, 5.77, 3.77 and 0.69, respectively, with the concentration significantly different. The positive control (commercial xylanase of *Trichoderma longibrachiatum)* resulted in reducing sugar extraction significantly similar than the one obtained by using *P. brevicompactum* MMS1910, *Aspergillus* sp. MMS1785 and *Penicillium* sp. MMS1986 (10.54 mM). The negative control without the enzyme resulted in the lowest concentration of reducing sugars (0.68 mM), significantly lower than every fungal extract except *C. ramotenellum* MMS1959.

**Figure 4:**
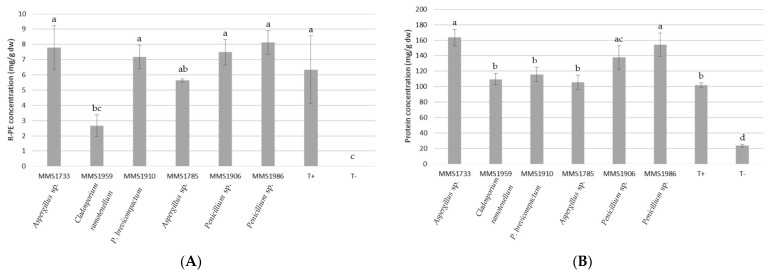
R-PE (**A**) and soluble protein (**B**) dosage from algal lysates after fungal enzymatic digestion (mg/g dw). R-PE concentration was determined according to the Sampath-Wiley and Neefus method [44]. Negative control (T-) corresponded to an algal lysate obtained with no enzyme added in the reactor, while positive control (T+) corresponded to an algal lysate obtained with addition of commercial xylanase in the reactor. Letters represent grouping of non-significantly different values according to post-hoc pair-wise Student’s test.

Figure 4 represents R-PE and total soluble protein releases obtained with all six fungal extracts. Determination of these releases was performed by using the spectrophotometric method [44] and bicinchoninic acid (BCA) assays, respectively, on fungal extracts obtained after the algal thallus degradation. These releases were compared with positive control (commercial xylanase) and negative control (no enzyme).

The algal lysates obtained using the enzymatic extract of *Penicillium* sp. MMS1986, *Aspergillus* sp. MMS1733, *Penicillium* sp. MMS1906 and *P. brevicompactum* MMS1910 seemed to be similarly characterized by the highest amounts of R-PE release, at 8.1, 7.8, 7.5 and 7.2 mg/g dw, respectively (Figure 4A). Algal degradation using *C. ramotenellum* MMS1959 enzymatic extract led to the lowest R-PE concentration in enzymatic reactor (2.67 mg/g dw), significantly different from the R-PE concentrations of the four previously mentioned strains (*p*-value < 0.01). The enzymatic extract of *Aspergillus* sp. MMS1785 allowed an intermediate R-PE release (5.63 mg/g dw). The positive control resulted in a R-PE release of 6.3 mg/g dw. This value was lower than the ones obtained by using fungal extracts of *Penicillium* sp. MMS1986, *Aspergillus* sp. MMS1733, *Penicillium* sp. MMS1906 and *P. brevicompactum* MMS1910 but not significantly different. It must be highlighted that this positive control was characterized by an important standard deviation. No R-PE release was obtained with the negative control without any enzyme. R-PE purity indexes of algal lysates ranged from 0.08 (*C. ramotenellum* MMS1959) to 0.25 (*P. brevicompactum* MMS1910). The fungal extract of *Aspergillus* sp. MMS1785, *Aspergillus* sp. MMS1733, *Penicillium* sp. MMS1906 and *Penicillium* sp. MMS1986 allowed us to obtain, respectively, R-PE purity indexes of algal lysates of 0.17, 0.20, 0.24 and 0.24.

The highest total protein recovery was obtained using the enzymatic extracts of *Aspergillus* sp. MMS1733 and *Penicillium* sp. MMS1986, with protein concentrations of 163.5 and 154.3 mg/g dw (Figure 4B). The use of enzymatic extracts of strains *Aspergillus* sp. MMS1785, *C. ramotenellum* MMS1959 and *P. brevicompactum* MMS1910 was characterized by low total protein contents in the lysate with concentrations of 105.5, 109.6 and 115.8 mg/g dw. Total protein contents obtained with these three strains were significantly different to the ones obtained with *Aspergillus* sp. MMS1733 and *Penicillium* sp. MMS1986 strains (*p*-value < 0.01). An intermediate total protein concentration in algal lysate of 138 mg/g dw was observed using MMS1906 enzymatic extract. The positive control resulted in a total protein release of 101.7 mg/g dw. This value was significantly lower than the ones obtained by using fungal extracts of *Aspergillus* sp. MMS1733, *Penicillium* sp. MMS1986 and *Penicillium* sp. MMS1906 (*p*-value < 0.01). The negative control without any enzyme resulted in a total protein release of 23.6 mg/g dw, significantly lower than the ones obtained by using fungal extracts (*p*-value < 0.001).

## 3. Discussion

### 3.1. Aspergillus, Penicillium and Cladosporium Marine Fungal Genera as Promising Xylanase and Cellulase Producers

The six fungal strains isolated and described according to their cellulolytic and xylanolytic potential, as well as to their ability to degrade the red seaweed *P. palmata* and to recover R-PE, belonged to the three fungal genera *Penicillium*, *Cladosporium* and *Aspergillus*. These fungal genera are widely distributed, notably in marine environments, as easily cultivable fungi. More specifically, *Aspergillus*, *Cladosporium* and *Penicillium* are dominant among all Ascomycota genera widely colonizing macroalgae, and they are characterized by a broad algal host specificity [7,45,46,47].

In a recent review, we have realized a state of the art on the polysaccharidase abilities of marine-derived fungi, focused on macroalgal polysaccharides [45]. Among various aptitudes in the degradation of macroalgal polysaccharides, fungal species belonging to *Aspergillus*, *Cladosporium* and *Penicillium* genera were recorded to exhibit both cellulolytic and xylanolytic activities [40,45,48,49].

### 3.2. Xylanolytic and Cellulolytic Profiles of Fungal Strains Related to Their Macroalgal Degradation Abilities and R-PE Recovery

Besides enzymatic assays applied on every six fungal strains, enzymatic extracts were then used for *P. palmata* biomass degradation. Extraction yield of reducing sugars, R-PE and total soluble proteins were then estimated. The R-PE concentration of macroalgal lysates were obtained according to the protocol developed by Sampath-Wiley et al. [44], optimized by Beer and Eshel [50].

Results obtained in the present study can be compared with previous ones obtained in our laboratory on *P. palmata* degradation with cellulolytic and xylanolytic enzymes. Regarding R-PE recovery, enzymatic assisted extraction without optimization resulted in up to 4 mg/g dw or 0.35 mg/g ww R-PE concentrations [24,32]. After optimization of the extraction protocol adapted for the use of commercial xylanase of *T. longibrachiatum*, the R-PE yield reached 12.36 mg/g dw [26]. In the present study, the highest R-PE yield reached 8.1 mg/g dw (*Penicillium* sp. MMS1986). Nevertheless, it is important to notice that the positive control used in the present study, expected to allow a 12.36 mg/g dw R-PE yield [26], resulted in a R-PE recovery almost twice lower, at only 6.3 mg/g dw. Enzymatic extracts of *Aspergillus* sp. MMS1733, *P. brevicompactum* MMS1910, *Penicillium* sp. MMS1906 and *Penicillium* sp. MMS1986 allowed a R-PE recovery higher than with commercial xylanase. The values of R-PE purity indexes of algal lysates were low using every fungal extract. These results could be explained because no purification processes were performed on algal lysates to improve R-PE purity. Moreover, these values are similar to the ones of a previous study without the purification process [26].

Regarding soluble protein recovery, previous enzymatic extraction without optimization led to protein yields below 2.5 mg/g ww [32]. After optimization of the extraction protocol adapted for the use of commercial xylanase, the soluble protein yield reached 30.58 mg/g dw [26]. In the present study, the positive control corresponding to this commercial *T. longibrachiatum* xylanase resulted in a protein recovery of 101.7 mg/g dw. This important gap between protein recovery values of the present study and the previous ones could be explained by dissimilarities in protocols used for protein assays. Indeed, protein assays of the present study were performed by using BCA methodology while the Bradford methodology was used in [26]. Interestingly, all fungal enzymatic extracts resulted in obtaining higher soluble protein yields compared to positive control, up to 163.5 mg/g dw for *Aspergillus* sp. MMS1733.

### 3.3. Biotechnological Potential of Fungal Enzymatic Extracts

It was previously mentioned that *Aspergillus* sp. MMS1785 and *Penicillium* sp. MMS1906 exhibited, respectively, the highest xylanase (978 U/mL) and cellulase (50.5 U/mL) activities. These two strains were then expected to allow a good macroalgal biomass degradation and consequently a good R-PE release, particularly regarding *Aspergillus* sp. MMS1785 because the *P. palmata* cell wall is mainly composed of xylan [18,19,34,35]. Nevertheless, while the enzymatic extract of *Aspergillus* sp. MMS1785 resulted in both high reducing sugar concentration (9.48 mM) and intermediate R-PE concentration (5.63 mg/g dw) in the macroalgal lysate compared with other strains as expected, this trend was reversed for *Penicillium* sp. MMS1906 enzymatic extract. Similarly, while the *P. palmata* degradation ability of *Aspergillus* sp. MMS1785 was also highlighted by the lowest total protein recovery (105 mg/g dw), *Penicillium* sp. MMS1906 was associated with a relatively high total protein concentration in the lysate, reaching 138 mg/g dw. These unexpected results could be explained by variability in the enzymatic substrate specificity of *Aspergillus* sp. MMS1785. Indeed, the nature of the substrate is known to greatly affect the efficiency of enzymes, notably marine fungal enzymes [51,52,53,54]. In the present study, wheat arabinoxylan was used as the substrate for DNS (dinitrosalicylic acid) assays because of its good water solubility and commercial availability, and its use resulted in the attribution of a high xylanase activity to *Aspergillus* sp. MMS1785. Then, considering the obtained results, the xylanase activity of *Aspergillus* sp. MMS1785 enzymatic extract should be lower when using *P. palmata* purified xylan than when using commercial wheat arabinoxylan. Interestingly, reducing sugar, R-PE and total protein concentrations seemed similar between *Aspergillus* sp. MMS1785 and the xylanase positive control. It is also possible that the low R-PE and total protein concentrations observed in the macroalgal lysate generated by the *Aspergillus* sp. MMS1785 enzymatic extract should be related to a potential proteolytic degradation. Indeed, enzymatic extracts were not purified, and proteases could putatively partially degrade *P. palmata* proteins, including R-PE [55,56,57,58]. With regard to the results for reducing sugars, several hypotheses can be put forward regarding the fact that the use of strain 1785 led to both a limited extraction of R-PE and proteins but a high concentration of reducing sugars in the algal lysate, and reversely for strain 1906. Although the degradation of xylan results in the production of xylose, and the assay of xylose can give an indication of the degree of algal degradation, it is important to note that fungi use xylose for their development. Each strain may therefore have a specific xylose requirement for its development. In addition, a standard incubation time of 7 days has been defined for enzyme production by the six fungal strains, but it is possible that they will not all be at the same level of development at the end of these 7 days.

Interestingly, opposite results were obtained considering *Aspergillus* sp. MMS1733. Similar to *C. ramotenellum* MMS1959, *Aspergillus* sp. MMS1733 exhibited particularly low cellulase and xylanase activities compared with other strains, at 295.4 and 18.2 U/mL, respectively. Nevertheless, the enzymatic extract of this strain allowed us to obtain high R-PE and protein concentrations. That could be explained by similar arguments explaining the results of *Aspergillus* sp. MMS1785. Indeed, contrarily to *Aspergillus* sp. MMS1785, *Aspergillus* sp. MMS1733 should have excreted xylanolytic and cellulolytic enzymes with low affinity for substrates used in DNS assays (wheat arabinoxylan and carboxymethyl cellulose, respectively), but with a great affinity with *P. palmata* polysaccharides. The high relative rate of protein recovery (including R-PE) could be explained by this highly specific enzymatic affinity, but also putatively by a low proteolytic activity. Nevertheless, the ratio (R-PE/total proteins) obtained with *Aspergillus* sp. MMS1733 did not seem particularly high compared with other isolates. Interestingly, both MMS1733 and MMS1785 are *Aspergillus* species. In the literature, several *Aspergillus* cellulase or xylanase were characterized as exhibiting high substrate specificities [59,60,61,62]. It could then be hypothesized that the present algicolous strains belonging to this genus would synthesize enzymatic arsenals with high substrate specificities.

The isolate *Penicillium* sp. MMS1986 was comparable with *Penicillium* sp. MMS1906, previously discussed. Total protein and R-PE concentrations obtained by using both enzymatic extracts were similar and among the highest compared with the other fungal isolates. Indeed, the use of both strains for these two assays showed results that were not significantly different. Moreover, *Penicillium* sp. MMS1986 was the fungal isolate which allowed the highest R-PE recovery (8.1 mg/g dw). Similarities between these two strains regarding their enzymatic strategies may be notably due to their taxonomic proximity. Several relative recent studies, realized on various genus including *Penicillium*, have demonstrated the relationship between genetic distance of fungal strains and their enzymatic profile [63,64,65,66]. Nevertheless, the natural habitats would also greatly influence the genome evolution and notably the enzymatic profile [64].

Compared with all the other fungal isolates, *C. ramotenellum* MMS1959 seemed to be the worse candidate for *P. palmata* degradation. As mentioned before, this isolate exhibited the lowest relative cellulase and xylanase activities and its use was characterized by the lowest reducing sugar and R-PE concentration. This strain might also excrete proteases potentially in part responsible for the low amount of recovered proteins. Among all the tested fungal strains, *C. ramotenellum* MMS1959 was the only one belonging to the *Cladosporium* genus. Further investigations on other *Cladosporium* isolates obtained from this macroalga may either allow descriptions of other various enzymatic profiles or result in the observation of a homogeneous enzymatic degradation strategy.

The strain *Penicillium brevicompactum* MMS1910 was the most promising considering its absolute enzymatic production. Indeed, it exhibited the second highest cellulase and xylanase activities. Nevertheless, the macroalgal degradation performed with this enzymatic extract did not seem of particular interest, resulting in a total soluble protein concentration quite low compared with the other fungi screened. However, it allowed a good R-PE recovery (7.2 mg/g dw), and the use of this fungal strain resulted in the best ratio (R-PE/total proteins), with R-PE corresponding to 6% of total protein content.

The use of fungal enzymatic extracts allowed better macroalgal degradation, R-PE or soluble protein recoveries compared to the commercial xylanase used as positive control. Thus, it seems that the xylanase activities from *P. palmata* associated fungal strains present a better efficiency and specificity compared with the *T. longibrachiatum*.

It would then be of high interest to further characterize the enzymatic composition of these fungal extracts beyond the quantification of both xylanolytic and cellulolytic activities. Moreover, optimization can be envisaged on the protocol of fungal culture by investigating conditions resulting potentially in a higher production of enzymes, such as incubation time, incubation temperature, humidity percentage or volume of conidial suspension. Optimization could also be performed on the protocol of macroalgal degradation and R-PE recovery, by modulating reaction parameters such as fungal enzymatic extract concentration, time and temperature of reaction, or amount of macroalgal biomass. It would also be interesting to finely characterize the level of cell wall polysaccharide using fungal extracts, to exhibit an eventual link between the level of degradation and the R-PE extraction rate. Indeed, it has been shown in this study that the consumption of xylose by fungi makes the measurement of reducing sugars insufficient for a detailed characterization of the parietal degradation of the algae. Moreover, in order to determine the polysaccharide profile and content in *P. palmata*, a procedure involving a first step of polysaccharide hydrolysis followed by a second step of high-performance liquid chromatography could be considered [67].

Even if the use of purified commercial enzymes is currently principally used in industry, the use of the extracted secretome can then permit, besides an economically viable deletion of purification procedure, to target complex substrates with the presence of several enzymes, potentially acting in synergy [42,68,69]. Optimization of the polysaccharide degradation and a reduced risk of contamination of the fungal culture medium by unwanted microorganisms can be performed by using fungal co-cultivation [41,69,70]. Because of the advantages given by the use of fungal co-cultivation on the enzymatic degradation of biological substrates [52], the industrial use of secretomes obtained from co-cultivation of the fungal strains investigated in the present study may be a promising way to investigate in the R-PE recovery from *P. palmata*. The strategy explored in this study, consisting of the use of fungi associated with the targeted alga in order to extract compounds of interest from this alga, could be tested and extended to red macroalgae other than *P. palmata*.

Other analyses of interest would consist of measurement and screening of potential proteolytic activities present in each fungal enzymatic extract and then resulting notably in partial R-PE degradation, as discussed above.

Several processes and methods of purification have already been explored and optimized for R-PE extracted from *P. palmata* [24,71]. The purification of R-PE from algal lysate would be necessary in order to consider eventual commercialization. In the same commercial perspective, it could be important to finely characterize and identify xylanases and cellulases of fungal extracts. Indeed, strategies of activity monitoring and of enzymatic biochemistry should be performed before an upscaling adapted to an eventual industrialization.

As previously mentioned, it seems to have a correlation between the enzymatic degrading abilities of *P. palmata* biomass degradation and the genetic assignation of fungal strains. Further investigations on other enzymatically active fungal strains isolated from *P. palmata* may be necessary to validate this hypothesis. This could also allow the investigation of the potential correlations between fungal isolation, incubation or environmental factors and cellulolytic and xylanolytic algicolous fungal abilities.

## 4. Material and Methods

### 4.1. Fungal Strains Isolation

*P. palmata* sampling was performed in the west coast of France at Valentin beach in Batz-sur-Mer (47.276222, −2.495004). The isolation of fungal isolates started with a wash of algal thalli three times with artificial sea water (ASW) which are then ground using a T25 Ultra-Turrax instrument (IKA^®^ Werke GmbH & Co. KG, Staufen, Germany) in ASW. The crushed material was then spread on solid culture media in 15 cm-diameter Petri dishes and incubated at 12 °C.

Two different media, the aspecific enriched Wickerham medium [72] and the specifically designed “*Palmaria palmata*” medium to mimic in situ conditions, were used for the incubation. Wickerham medium composition was glucose 10 g/L (Rectapur, VWR-Avantor, Rosny-sous-Bois, France), yeast extract 3 g/L, malt extract 3 g/L, agar 17 g/L (Condalab Madrid, Spain), peptone 5 g/L, (Biokar Diagnostics, Beauvais, France), sea salts 30 g/L (Reef Crystals, Aquarium Systems, Strasbourg, France), chloramphenicol 0.5 g/L (Merck Chimie, Fontenay sous Bois, France) and penicillin G sodium salt 0.2 g/L (Sigma-Aldrich, Saint-Quentin-Fallavier, France). Specific “*Palmaria palmata*” sterile medium consisted of *P. palmata* lyophilized powder 15.6 g/L, agar 17 g/L, sea salts 30 g/L, chloramphenicol 0.5 g/L and penicillin G 0.2 g/L.

Fungal colonies that grew on the inoculated plates were isolated under sterile conditions on DCA medium (dextrose 40 g/L, enzymatic digest of casein 10 g/L, agar 15 g/L, Condalab, Madrid, Spain) and incubated at 12 °C. Each isolate was then subcultured and stored at 4 °C and added to the laboratory fungal collection. The subcultured isolate was incubated at 12 °C and was observed as mentioned later after growth.

### 4.2. Fungal Strain Identification

Fungal identification was first performed on the basis of macroscopic and microscopic morphological features leading to an identification at the genus level when conidiophores were visible. Complementary identifications were made using a molecular approach. The DNA of all the fungal isolates was extracted using the FastDNA Spin Kit (MP Biomedicals, Illkirch, France) following the manufacturer’s recommendations and the extracted DNA concentration was measured with a NanoDrop spectrophotometer (Thermo Fisher Scientific, Illkirch, France) and standardized at 7.5 ng/µL. DNA extracts were stored at −20 °C.

The internal transcribed spacers (ITS1 and 2) were chosen to sequence the fungal isolates. Primers used for the amplification were ITS4 (5′-TCCTCCGCTTATTGATATGC-3′) and ITS5 (5′-GGAAGTAAAAGTCGTAACAAGG-3′), in forward and reverse position, respectively. The PCR reaction was performed on 10 ng of DNA for each isolate with 25 µL mix containing 1X of PCR buffer, 2 mM of MgCl_2_, 0.2 µM of both ITS4 and ITS5 primers (Thermo Fisher Scientifics), 0.2 mM of dNTP, 0.025 U/µL of Taq Polymerase (Promega). The amplification consisted of an initial denaturation step at 95 °C for 5 min, followed by 29 cycles of 60 s at 95 °C, 60 s at 55 °C and 60 s at 72 °C, and a final elongation step at 72 °C for 10 min. PCR products were sent to Eurofins Scientific (Ebersberg, Germany) for sequencing using ITS4 and ITS5 primers. For each fungal strain, both forward and reverse sequences were aligned using the de novo process using Geneious software (2021.2.2 version, www.geneious.com (accessed on 24 August 2021)) to design a consensus sequence. The consensus sequences were pulled in the Blast function of the NCBI database (default algorithm: max target sequences: 100; expect threshold: 0.05; word size: 28; max matches in a query range: 0; match/mismatch scores: 1, −2; gap costs: linear; filter: low complexity region; mask: mask for lookup table only), and assignations were then performed for each fungal isolate. Based on ITS markers, the assignation of several fungal isolates was limited to the genus level and supplementary analyses were performed using other gene markers in order to obtain a better accuracy.

The PCR reaction was performed on 40 ng and 15 ng of genomic DNA for actin and β-tubulin sequencing, respectively. The PCR reaction for actin sequencing was performed in 25 µL mix, containing 1X of PCR buffer, 2 mM of MgCl_2_, 0.5 µM of both ACT-512F and ACT-783R primers (Thermo Fisher Scientifics), 0.2 mM of dNTP, 0.05 U/µL of Taq Polymerase and 40 ng of genomic DNA. The amplification consisted of an initial denaturation step at 95 °C for 8 min, followed by 29 cycles of 15 s at 95 °C, 20 s at 58 °C and 60 s at 72 °C, and a final elongation step at 72 °C for 5 min. The PCR reaction for β-tubulin sequencing was performed in 25 µL mix, containing 1X of PCR buffer, 1.5 mM of MgCl_2_, 0.2 µM of both Bt2a and Bt2b primers (Thermo Fisher Scientifics), 0.2 mM of dNTP, 0.025 U/µL of Taq Polymerase and 10 ng of genomic DNA. The amplification consisted of an initial denaturation step at 94 °C for 5 min, followed by 35 cycles of 60 s at 94 °C, 60 s at 61 °C and 60 s at 72 °C, and a final elongation step at 72 °C for 5 min. PCR products were sent to Eurofins Scientific (Ebersberg, Germany) for sequencing using corresponding primers and both forward and reverse sequences were aligned using the de novo process and Geneious software to design a consensus sequence. All fungal assignations were performed on the basis of the best hit displaying query coverage and identity higher than 99% or on the highest values when the best match was lower.

Fungal gene sequences used for assignations were deposited into Genbank. Their accession numbers and all informative data regarding the six fungal strains isolated from *P. palmata* are summarized in Table 1.

### 4.3. Strain Cultivation and Preparation of Conidia Suspension

Fungal strains were grown in a 50 mL conical flask on 15–20 mL of DCA medium. After 14 days of incubation at 27 °C, a conidial suspension was prepared with the addition of 5 mL of sterile distilled water and 0.1 mL of Tween 80 in the tube of fungal growth. Conidial suspensions were standardized at 10^6^ conidia/mL and were then aliquoted and stored at −20 °C.

### 4.4. Enzyme Production

A sterile culture medium based on lyophilized powder of *P. palmata* at 80% humidity was prepared for fungal enzyme production. Similar fungal culture media at 80% humidity have already been used for enzymatic production, with various carbon sources [73,74]. For fungal growth and enzymatic production, 1.5 mL of conidial suspension was added on all the surface of culture medium prepared with 20 g of lyophilized powder of *P. palmata*. Such culture was performed in triplicate for each strain and incubated 7 days at 27 °C. Biological negative control was performed with the incubation of culture medium without any conidia suspension. After incubation, 35 mL of sterile distilled water was added in the fungal cultures and homogenization was performed using a blender (Virtis 23, New York, NY, USA). The mash was then centrifuged 15 min at 9500 rpm (SL 8R, Thermo Scientific^TM^, Waltham, MA, USA) and the supernatant was filtered through a glass microfiber filter at 1.2 µm retention porosity (Whatman^TM^, Buckinghamshire, United Kingdom) using a vacuum pump (Millipore, Burlington, VT, USA). Glycerol 87% *w*/*w* (PlusOne, St George, UT, USA) was added to reach a final concentration of 10% in the filtrate which is then stored at −80 °C. This filtrate corresponded to the enzymatic extract used for the following tests.

### 4.5. Protein Quantification

Protein quantifications were performed using the BCA method with the Pierce^TM^ BCA Protein Assay Kit (Thermo Scientific^TM^) according to the manufacturer’s instructions. Measurements were performed on the ELx800 UV Universal Microplate Reader (BioTek, Winooski, VT, USA).

### 4.6. Enzymatic Assays

Enzymatic assays were performed on enzymatic extracts to determine both cellulase and xylanase activities of every isolated fungal strain, using the DNS method [75]. Briefly, this method allows the measurement of reducing sugar production by enzymatic extract from a substrate of sodium carboxymethyl cellulose (Sigma-Aldrich, St. Louis, MO, USA) and wheat arabinoxylan (Megazyme, Wicklow, Ireland) for cellulase- and xylanase-assays, respectively. Both substrates, sodium carboxymethyl cellulose and arabinoxylan, were prepared at 1% in 0.05 M pH 5.3 sodium citrate buffer (citric acid 21 g/L, Acros Organics, Geel, Belgium, and sodium citrate tribasic dihydrate 29.6 g/L, Sigma-Aldrich, St. Louis, MO, USA). A solution of DNS 1% was prepared (DNS, Sigma-Aldrich, St. Louis, MO, USA, 1%; Phenol, Fluka^TM^, Honeywell, Charlotte, NC, USA, 0.2%; sodium sulphite, Sigma-Aldrich, 0.05%; NaOH, Fluka^TM^, 1%). Commercial enzymatic solutions were used as control, being solutions of 1% cellulase and 1% xylanase from *T. longibrachiatum* (Sigma-Aldrich, St. Louis, MO, USA). Reaction consisted of mixing 1.8 mL of substrate solution with 200 µL of enzymatic solution (fungal extract or control), heating this mix at 50 °C during 5 min, adding 3 mL of 1% DNS solution, heating the mix at 100 °C during 15 min, and reading the OD at 540 nm (Shimadzu, Kyoto, Japan) after a cooling step of tubes in cold water. One unit of enzyme activity was defined as the amount of enzyme allowing the release of 1 µmol of product (xylose for xylanase and glucose for cellulase) per minute in the assay condition.

### 4.7. R-PE Extraction from P. palmata

In a previous study, commercial xylanase powder concentration was defined as optimal at 17.8 g/kg dw (35.6 mg in 200 mL of solution of reaction) [26]. This commercial extract was used at this concentration for positive control. To standardize the R-PE extraction conditions for the six isolated fungal strains, a volume of enzymatic extract allowing a xylanase activity equivalent to 35.6 mg of commercial xylanase was used. These volumes were established using results of DNS assays realized on arabinoxylan as the substrate and described in Table 2.

The R-PE extraction from *P. palmata* was performed on thalli sampled the 14 July 2021 and stored at −20 °C after washing. The reaction consisted of the degradation of 15 g of cut fresh thalli of *P. palmata* occurring in an acetate buffer 50 mM pH 5 (anhydrous acetic acid, Thermo Scientific; sodium acetate, Sigma-Aldrich) with the addition of either 35.6 mg of commercial xylanase or corresponding volume of enzymatic extract (Table 2), in a total volume of 200 mL in 200 mL enzymatic reactors (Fisher Scientific, Wheaton, IL, USA). The reaction was performed during 320 min at 24 °C with Polystat 36 (Fisher Scientific), in darkness and with an agitation of 250 rpm (Biostir^®^ Wheaton, Millville, NJ, USA) [26]. Solution was then roughly filtered through a fine sieve and was centrifuged 20 min at 4 °C and 9500 rpm (SL 8R, Thermo Scientific). The supernatant corresponding to R-PE extract was aliquoted and stored at −20 °C.

### 4.8. Dosage of R-PE, Proteins and Reducing Sugars

Algal lysates obtained after *P. palmata* enzymatic degradation were used to determine reducing sugar, R-PE and protein concentrations using the following protocols. Reducing sugar was assayed using the DNS method as mentioned above but without the first step of enzymatic incubation. The concentration of reducing sugars in the fungal extracts was deduced from that in the algal lysates, in order to determine the concentration only due to hydrolysis of the algal polysaccharides. R-PE assays were performed by using the spectrophotometric method and the OD measurements were performed using a Shimadzu UV1900 spectrophotometer (Shimadzu, Kyoto, Japan). The following formula was used to obtain the R-PE concentration in mg/mL: [R-PE] = 0.1247 [(*A*_564_ − *A*_730_) − 0.4583 (*A*_0618_ − *A*_730_)] [44]. The *A*_565nm_*/A*_280nm_ ratio was used on algal lysates to determine the R-PE PI [26]. Data of R-PE and soluble protein concentrations initially expressed in mg/mL were converted in mg/g dw based on the previously mentioned equivalence 1 g wet alga = 0.15 g dry alga.

### 4.9. Statistical Analysis

Statistical analyses were performed using Rstudio (RStudio Team, 2023.06.0+421 version) and R software (R Core Team, 4.2.3 version). The significances of differences between values of enzymatic activities, specific activities, reducing sugar, R-PE and total soluble protein concentrations were established by using a pairwise Student’s test from the rstatix package [76]. This test was the only solution to test the equality of expected results and not the stochastic equality. Similarity between strains is highlighted on the graphs with similar letters for similar data (*p*-value ≤ 0.05).

## 5. Conclusions

In conclusion, to the best of our knowledge, this is the first study on the use of enzymes of *P. palmata* associated fungi in the degradation of its own biomass for biomolecules recovery. The present study has resulted in the quantification of both cellulolytic and xylanolytic activities of six fungal isolates derived from *P. palmata*. The fungal culture process using only *P. palmata* biomass as the substrate succeeded in the production of enzymatically active fungal secretomes. The use of these fungal secretomes showed results of high interest in the cell wall degradation and the R-PE recovery. Several strains seemed more promising than the others such as *Penicillium* sp. MMS1906, *Aspergillus* sp. MMS1733 and *Penicillium brevicompactum* MMS1910. Interestingly, the most promising isolates in terms of R-PE recovery did not necessarily correspond to the isolates characterized by the highest xylanolytic and cellulolytic activities. Further work and investigations are required notably in the optimization of the uses of fungal secretomes in the R-PE recovery from *P. palmata*.

## Figures and Tables

**Table 1:** Strains isolated from *P. palmata* and studied for the enzymatic production. Molecular markers used for the identification and culture medium used for the isolation were indicated.

Fungal Isolate	Assignation	Molecular Marker	Isolation Culture Medium	Genbank Accession Numbers	BLASTn NCBI	
					Identity (%)	Query Cover (%)
MMS1733	*Aspergillus* sp.	ITS	Palm	ON217540	100	100
MMS1785	*Aspergillus* sp.	ITS	Palm	ON217539	100	100
MMS1906	*Penicillium* sp.	ITS	Wick	ON331769	100	100
MMS1910	*Penicillium brevicompactum*	ITS/β-tubulin	Wick	ON217538/ON217535	100/100	100/100
MMS1959	*Cladosporium ramotenellum*	ITS/actin	Palm	ON217537/ON209120	100/100	100/100
MMS1986	*Penicillium* sp.	ITS	Palm	ON331783	100	100

**Table 2:** Composition of reaction solution of R-PE extraction for each fungal strain.

Fungal Isolate	Volume of Fungal Extract (mL)	Volume of Acetate Buffer (mL)	Cut *P. palmata* Thalli (g)
*Aspergillus* sp. MMS1733	44.5	155.5	15
*Aspergillus* sp. MMS1785	14	186	15
*Penicillium* sp. MMS1906	32.3	167.7	15
*Penicillium brevicompactum* MMS1910	16.6	183.4	15
*Cladosporium ramotenellum* MMS1959	39.5	160.5	15
*Penicillium* sp. MMS1986	29.6	170.4	15
Positive control	35.6 mg *	200	15

* Mass of commercial xylanase powder from *T. longibrachiatum*.

## Data Availability

ITS, β-tubulin and actin sequences of fungal strains are available in Genbank (https://www.ncbi.nlm.nih.gov/genbank/ (accessed on 21 June 2023)), using their accession numbers.
